# Calreticulin-Mediated Quality Control of the Non-Classical MHC-I Molecule MICA: Implications for Immune Surveillance

**DOI:** 10.3390/ijms27031310

**Published:** 2026-01-28

**Authors:** Fabiola González-Herrera, Karen Toledo-Stuardo, Antonio E. Serrano, Marcela Gatica-Andrades, Douglas J. Matthies, Valentina López, Ignacio Aguayo, Sebastián Indo, María José Garrido, Yuneisy Guerra, Samantha Tello, Ivo Campos, Flavio Salazar-Onfray, Gerald Zapata-Torres, Carolina H. Ribeiro, María Carmen Molina

**Affiliations:** 1 Núcleo Interdisciplinario de Farmacología e Inmunología, Instituto de Ciencias Biomédicas (ICBM), Facultad de Medicina, Universidad de Chile, Santiago 8380453, Chile; fabiola.gonzalez@ug.uchile.cl (F.G.-H.); karen.toledo.stuardo@gmail.com (K.T.-S.); dmatthies@gmail.com (D.J.M.); vlopezg@ug.uchile.cl (V.L.); ignacio.aguayo.s@ug.uchile.cl (I.A.); maria.garridog@utem.cl (M.J.G.); yuneisy.guerra@ug.uchile.cl (Y.G.); samantha.tello.a@gmail.com (S.T.); ivo.campos@ug.uchile.cl (I.C.); fsalazar@uchile.cl (F.S.-O.); chager@med.uchile.cl (C.H.R.); 2 Bioicus Lab, Oxfordshire OX11 6AF, UK; antonio@bioicus.com; 3 Facultad de Ciencias de la Salud, Universidad del Alba, La Serena 1710555, Chile; marcela.gatica@udalba.cl; 4 Center for Molecular Modeling, Biophysics and Bioinformatics (CM2B2), Faculty of Chemical and Pharmaceutical Sciences, University of Chile, Santiago 8380494, Chile; gzapata@uchile.cl; 5 Department of Basic and Clinical Oncology, Faculty of Medicine, University of Chile, Santiago 8380453, Chile; srindo@uchile.cl; 6 Millennium Institute for Immunology and Immunotherapy, Faculty of Medicine, University of Chile, Santiago 8380453, Chile

**Keywords:** MICA, calreticulin, endoplasmic reticulum quality control, NKG2D ligands, melanoma, glycosylation, immune surveillance

## Abstract

Major histocompatibility complex class I chain-related gene A (MICA) is a non-classical MHC-I molecule essential for immune surveillance, yet its intracellular maturation remains poorly understood. We show that MICA is predominantly retained intracellularly in melanoma cells and colocalizes with the endoplasmic reticulum chaperone calreticulin (CRT). Notably, MICA also colocalizes with CRT in healthy skin. Immunoprecipitation assays reveal that CRT preferentially associates with a low-molecular-weight form of MICA. Recombinant protein assays and in silico analyses support direct interaction between CRT and non-glycosylated MICA, but not with fully glycosylated eukaryotic MICA. These findings identify CRT-dependent retention of MICA as a physiological checkpoint that may be dysregulated in melanoma to promote immune evasion.

## 1. Introduction

Class I major histocompatibility complex (MHC-I) molecules are essential for immune surveillance, allowing cytotoxic lymphocytes to detect and eliminate infected or transformed cells [1]. Their biogenesis and surface expression rely on a strict quality control system in the endoplasmic reticulum (ER), where chaperones and folding enzymes coordinate the correct maturation of the heavy chain-β_2_-microglobulin heterodimer and the peptide loading required for its stability prior to membrane export [2]. In this process, the calreticulin/calnexin (CRT/CNX) cycle plays a central role by recognizing monoglucosylated N-glycans and retaining glycoproteins that have not yet reached their native conformation or display immature glycosylation, ensuring that only fully folded and peptide-loaded MHC-I complexes are exported to the cell surface [3]. Importantly, increasing evidence indicates that the immunological functions of MHC molecules extend beyond peptide presentation, involving additional layers of post-translational regulation that remain incompletely understood at the molecular level.

In addition to classical MHC-I molecules, the immune system includes non-classical MHC-I molecules. Among them, the MHC class I-related chains A and B (MICA and MICB, respectively) are stress-induced glycoproteins that function as ligands for the cytotoxicity-activating receptor NKG2D on NK cells and CD8^+^ T lymphocytes [4,5]. Unlike classical MHC-I molecules, MICA and MICB do not bind peptides or β_2_-microglobulin [6], placing them outside the canonical paradigm of antigen presentation that has historically dominated MHC research, and suggesting that their folding, maturation, and intracellular trafficking may depend on distinct or modified quality control pathways [7]. However, the mechanisms governing the biogenesis of these non-classical MHC-I molecules remain poorly characterized, despite their relevance in tumor immunosurveillance and immune evasion [7,8]. Understanding the molecular regulation of such non-classical MHC molecules is increasingly recognized as critical for deciphering MHC-linked immune pathologies beyond transplantation and autoimmunity.

The aberrant expression or intracellular retention of NKG2D ligands is a well-documented mechanism by which tumors, such as melanoma, reduce their immunogenicity [7,9]. Several studies have reported that immature or under-glycosylated forms of MICA are retained in intracellular compartments and degraded, thereby limiting their surface expression and attenuating NK-cell activation [9,10,11]. Nevertheless, the specific ER chaperones and quality control elements responsible for this retention remain unclear, representing a gap in the molecular understanding of how MHC-related immune modulation is regulated in cancer.

CRT is a lectin chaperone residing in the ER that participates in the folding of a vast repertoire of glycoproteins [3,12]. Beyond its canonical role in the CRT/CNX cycle, CRT can act as a retention factor for mutated, misfolded, or improperly glycosylated proteins, thereby modulating their intracellular trafficking and fate [3,13]. Given its established role in the quality control of classical MHC-I molecules, CRT emerges as a strong candidate regulator of non-classical MHC-I-related proteins, such as MICA and MICB [1,2]. Since these ligands are glycoproteins that require complex post-translational modifications for stability and expression [10,11], the possibility arises that CRT contributes to their quality control [3,12], directly influencing their availability at the cell surface and, consequently, their immunological function [9].

Although the intracellular retention of MICA has been described as a tumor immune-evasion mechanism [9], it remains unknown which components of the ER quality control machinery participate in this process and whether such interactions also occur in non-transformed tissues. This conceptual gap raises the possibility that the interaction between CRT and MICA represents a physiological step in ER quality control that may become dysregulated in the tumor context.

In this study, we investigated whether CRT interacts with MICA during its maturation in the ER and whether this interaction depends on MICA glycosylation state. Using skin malignant melanoma biopsies, healthy skin tissue samples, the human melanoma BL cell line, and recombinant prokaryotic and eukaryotic systems, we analyzed the subcellular localization of MICA/MICB, their colocalization with ER chaperones, and the capacity of CRT to associate with differentially glycosylated forms of MICA. Our results reveal that CRT preferentially associates with a low-molecular-weight variant of MICA, identified as a less-glycosylated variant, and that this interaction can occur when MICA lacks complex glycan modifications. These findings suggest that CRT plays a central role in the quality control of MICA and proposes a mechanism through which the biogenesis of non-classical MHC-I molecules may influence tumor immunosurveillance.

## 2. Results

### 2.1. MICA and MICB Are Predominantly Retained Intracellularly in Malignant Melanoma

**Figure 1:**
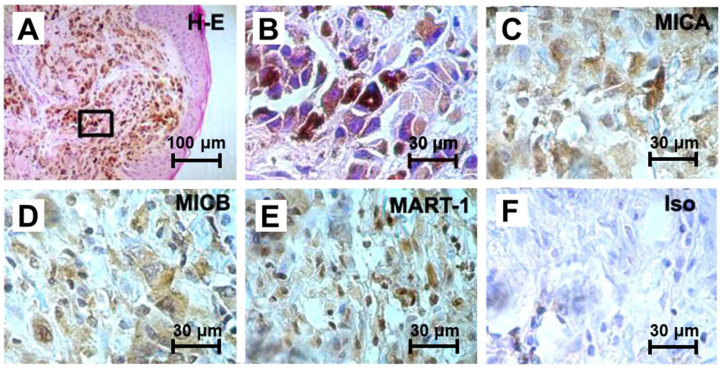
MICA and MICB Expression in Malignant Skin Melanoma Tissue. Biopsy samples of advanced cutaneous melanoma were paraffin-embedded, sectioned at 4 μm, and stained with hematoxilin–eosin (H-E) (**A**,**B**). Deparaffinized and ethanol-rehydrated sections were incubated with anti-MICA antibody (**C**), anti-MICB antibody (**D**), anti-MART-1 antibody (**E**), and IgG2b as an isotype control (**F**). Brown color indicates HRP-DAB staining, contrasted with blue nuclear hematoxylin. Representative images from two independent biopsies are shown. Scale bars represent 100 and 30 μm, as indicated.

To determine the expression of MICA and MICB in malignant skin melanoma, immunohistochemical staining was performed on paraffin-embedded melanoma tissue sections. Representative photomicrographs are shown in Figure 1. Hematoxylin-eosin staining delineated the histological regions analyzed (Figure 1A,B). Both MICA (Figure 1C) and MICB (Figure 1D) displayed a prominent cytoplasmic distribution in tumor cells. MART-1, a well-established melanoma-associated antigen, was used as a positive control (Figure 1E), while isotype-matched antibodies served as negative controls (Figure 1F). These results indicate that skin melanoma cells express MICA and MICB mainly in intracellular compartments.

### 2.2. MICA and MICB Colocalize with Endoplasmic Reticulum Chaperones in Melanoma Cells

**Figure 2:**
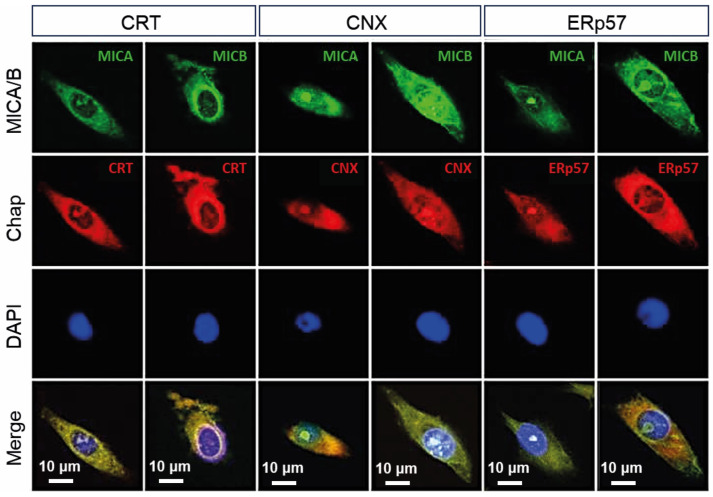
MICA and MICB are expressed in the cytoplasm of BL melanoma cells and colocalize with ER chaperones. Cells were seeded on coverslips, fixed, and subsequently incubated with specific primary antibodies and fluorophore-conjugated secondary antibodies. Confocal microscopy images of BL cells reveal MICA or MICB (in green) co-localizing with chaperone molecules calreticulin (CRT), calnexin (CNX), and ERp57 (in red). The final overlay (yellow) includes DAPI as a nuclear marker (blue). Representative images from three independent experiments are shown. Scale bars represent 10 μm.

The subcellular localization of MICA and MICB was next analyzed in the BL melanoma cell line using immunofluorescence and confocal microscopy. Both NKG2D ligands were detected intracellularly and exhibited similar distribution patterns (Figure 2). Staining of ER chaperones revealed overlapping fluorescence signals between MICA or MICB and CRT, CNX, and ERp57 (merged panels), consistent with partial localization within the ER network. The staining pattern was predominantly perinuclear, typical of ER distribution, and no defined plasma membrane signal was detected under the experimental conditions used. Occasional signals adjacent to the nucleus were observed; given the diffraction-limited resolution of confocal microscopy, these signals were interpreted as perinuclear rather than intranuclear localization.

Negative control samples incubated with Alexa Fluor 488-conjugated IgG2b antibody and preimmune normal rabbit serum (RS) conjugated to Alexa Fluor 647 showed no detectable labeling (Supplementary Figure S1).

### 2.3. Calreticulin Associates with MICA in Melanoma Cells

Given the high coding sequence homology between MICA and MICB (91%; [14]) and their similar intracellular localization patterns (Supplementary Figure S2), subsequent experiments focused on MICA as a representative NKG2D ligand. Because CRT is known to associate with MHC-I molecules during ER folding [15], we investigated whether a similar interaction occurs between CRT and MICA in melanoma cells.

**Figure 3:**
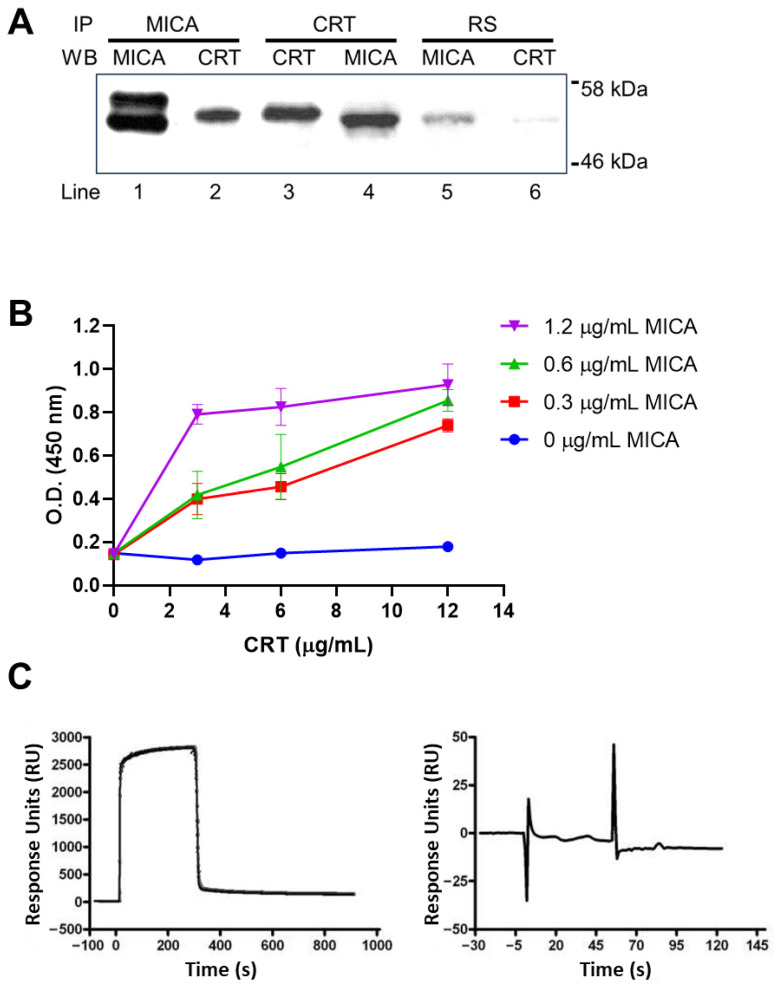
CRT associates with MICA and preferentially binds immature forms of the ligand. (**A**) Co-immunoprecipitation of CRT with MICA in BL melanoma cells. CRT preferentially associates with the lower-molecular-weight form of MICA. A representative blot from five independent experiments is shown. (**B**) ELISA analysis showing direct binding of recombinant CRT to recombinant MICA produced in a prokaryotic system. Data represents mean values ± standard deviation (SD) of three independent experiments. (**C**) SPR analysis showing no detectable interaction between CRT and recombinant MICA derived from HEK-293T cells. A representative sensorgram obtained using 1 nM CRT and 1 μg/μL MICA is shown (right plot). The Ephrin B2 receptor–sialic acid interaction was used as a positive control (left plot).

BL melanoma cells were lysed and immunoprecipitated with antibodies specific to MICA or CRT, with RS as a negative control. Immunoprecipitation of MICA revealed two bands upon immunoblot analysis, differing by approximately 7 kDa (Figure 3A, lane 1). Notably, CRT was detected in MICA immunoprecipitates (Figure 3A, lane 2), suggesting an association between these proteins. Reciprocal immunoprecipitation using anti-CRT antibodies followed by probing with anti-MICA antibodies confirmed this interaction (Figure 3A, lane 4). Importantly, CRT preferentially co-precipitated with the lower-molecular-weight form of MICA, while the higher-molecular-weight band was less represented.

The presence of two MICA species is consistent with heterogeneous N-glycosylation states, suggesting that CRT preferentially associates with underglycosylated or immature forms of MICA within the ER. Collectively, these results indicate that MICA and CRT form a complex in melanoma cells, supporting a role for CRT in the quality-control pathway of MICA.

### 2.4. Direct Interaction Between MICA and Calreticulin May Be Influenced by the Glycosylation State of MICA

After demonstrating the association between endogenous MICA and CRT in BL melanoma cells, we next assessed whether this interaction could also occur using recombinant proteins. ELISA-based assays using recombinant human MICA and CRT produced in a prokaryotic expression system revealed a dose-dependent increase in CRT binding to immobilized MICA (Figure 3B), indicating a direct molecular association under non-glycosylated conditions.

To evaluate whether this interaction was preserved when MICA was expressed in a eukaryotic system, the extracellular domain of MICA was produced in HEK-293T cells and analyzed by surface plasmon resonance (SPR). In contrast to the ELISA results, no detectable interaction between recombinant CRT and fully glycosylated eukaryotic MICA was observed at any of the tested concentrations (1–20 nM) (Figure 3C, right, representative sensorgram shown). Importantly, the SPR performance was confirmed by a robust interaction between the Ephrin B2 receptor and the sialic acid complex used as a positive control in a parallel flow cell (Figure 3C, left).

Together, these results indicate that the interaction between CRT and MICA is strongly influenced by MICA glycosylation state, with preferential binding to non-glycosylated forms.

### 2.5. MICA and Calreticulin Partially Colocalize in Healthy Skin Tissue

**Figure 4:**
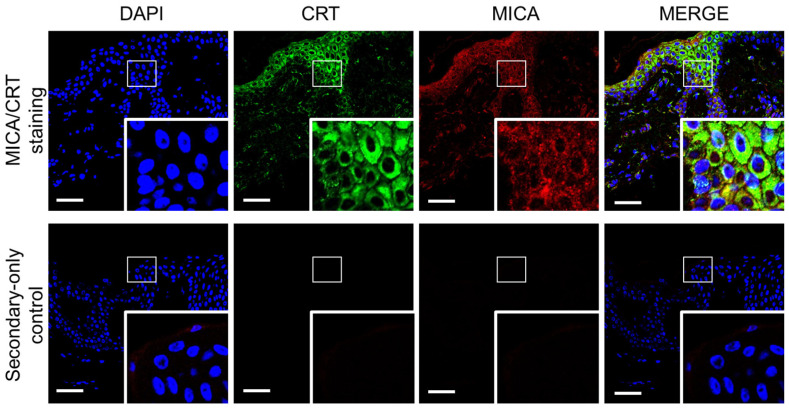
Expression and partial colocalization of MICA and CRT in healthy skin tissue. Immunofluorescence images of a paraffin-embedded healthy human skin tissue sample stained for CRT (green), MICA (red), and nuclei (DAPI, blue). The upper row shows samples incubated with primary and secondary antibodies, while the lower row shows negative controls incubated with secondary antibodies only. Insets indicate magnified regions. Channel merging was performed for qualitative visualization of colocalization. Representative images from two independent biopsies are shown. Scale bars represent 50 μm.

To determine whether the association between MICA and CRT also occurs under physiological conditions, immunofluorescence analysis was performed on healthy human skin tissue. Low-level MICA expression was detected and primarily localized to basal keratinocytes and skin appendages (Figure 4). In contrast, CRT exhibited a widespread intracellular distribution consistent with its known localization in the ER of keratinocytes. Merged images revealed partial colocalization between MICA and CRT in selected regions, particularly within subsets of basal epidermal keratinocytes.

Negative control sections incubated with secondary antibodies alone showed no specific signal neither for MICA nor CRT, confirming the staining specificity (Figure 4, lower panels). Colocalization was assessed qualitatively based on channel merging. These findings indicate that MICA and CRT can also colocalize in non-malignant tissue, supporting the notion that their association reflects a physiological quality-control process rather than an exclusively tumor-associated mechanism.

### 2.6. In Silico Modeling Supports a Plausible Interaction Interface Between MICA and Calreticulin

**Figure 5:**
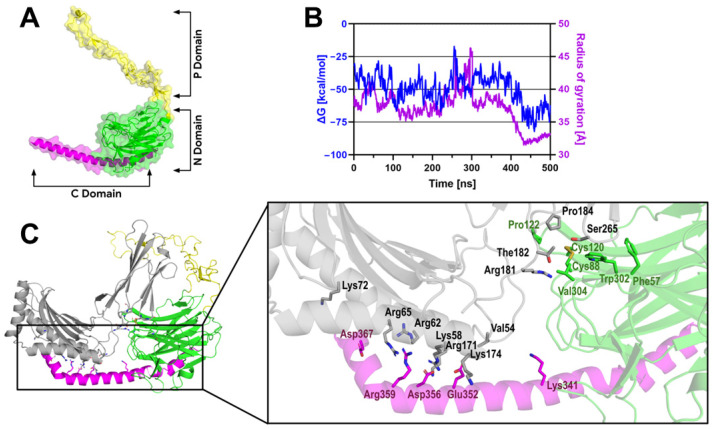
Structural stability of the MICA-CRT complex throughout molecular dynamics. (**A**) Three-dimensional representation of CRT showing the P domain in yellow, the N domain in green, and the C domain in magenta. The structure is shown in combined surface and cartoon renderings. (**B**) Rg of the complex (purple), demonstrating a sharp transition toward a compact state after 400 ns of simulation. Binding free energy calculated via MM/GBSA (blue). The energy profile reveals a significant thermodynamic stabilization (reaching ~−75 kcal/mol) that correlates with the structural compaction of the Rg. The cartoon displayed in (**C**) represents MICA-CRT complex structure. MICA protein is shown in gray. The right panel shows a zoomed-in view of the interaction interface between MICA (gray) and the N-domain (green) and C-domain (magenta) of CRT. Key amino acid residues contributing significantly to the binding energy, as determined by per-residue binding energy decomposition, are represented as stick models and labeled with their residue type and position.

To elucidate the structural basis of MICA-CRT interaction, homology models were generated for both proteins. The CRT protein structure, highlighting the P and N domains, is shown in Figure 5A, and a representative complex is presented in Figure 5C. The resulting structures were subjected to molecular docking to predict potential binding poses of the complex. From the docking results, the three most populated clusters were selected to generate the initial systems for Molecular Dynamics (MD) simulations. Following preliminary MD sampling and MM/GBSA assessment, the most energetically stable complex was identified and subjected to an in-depth conformational and thermodynamic analysis.

The MD trajectory shows that the MICA-CRT complex undergoes a significant conformational change. Analysis of the RMSD demonstrates that, although the global complex appears highly unstable, fluctuating between 15 and 20 Å. This behavior is driven primarily by the intrinsic disorder of the CRT P-domain (Supplementary Figure S4). When the P-domain is excluded from the calculation, the structural core of the complex stabilizes below 10 Å, reaching a steady plateau after 400 ns. The RMSF profiles further illustrate this modular flexibility, showing that the CRT N-domain and C-domain maintain high structural integrity with fluctuations consistently below 2 Å, whereas the P-domain acts as a flexible arm with fluctuations peaking at approximately 30 Å (Supplementary Figure S4). In contrast, the MHC-like domains of MICA (α1, α2, and α3) exhibit rigid-body behavior, with internal RMSD values stabilizing near 6 Å.

A critical transition in the binding landscape is observed between 400 and 430 ns, where the Radius of Gyration (Rg) undergoes a sharp contraction from an expanded state of approximately 40 Å to a compact state of approximately 32 Å (Figure 5B). This structural collapse correlates precisely with a drastic enhancement in binding affinity, as the ΔG_MM/GBSA_ evolves from a fluctuating state of approximately −40 kcal/mol to a robustly stabilized minimum near −72 kcal/mol in the final 70 ns (Figure 5B). This thermodynamic “locking” suggests that the initial phase of the simulation involved an extensive conformational search by the flexible CRT domains, culminating in a highly packed, energetically favorable state. The Dynamic Cross-Correlation Matrix (DCCM) validates this coupling, revealing a strong positive correlation between the CRT core and the MICA α1/α2 interface (Supplementary Figure S4).

Per-residue energy decomposition identifies the specific chemical drivers of this association, anchoring the interaction through a cluster of high-contribution residues, shown in Figure 5C. Collectively, these data provide compelling evidence for the viability of the MICA-CRT complex, in which the P-domain facilitates a conformational search that ultimately leads to a collapsed, high-affinity state characterized by significant compactness and a stable interfacial electrostatic network.

## 3. Discussion

This study defines a previously underappreciated connection between the non-classical MHC class I molecule MICA and the ER chaperone CRT, highlighting ER quality-control mechanisms as key regulators of MICA maturation and intracellular trafficking. By focusing on a non-classical MHC-I-related molecule that does not participate in antigen presentation, our findings expand the current view of MHC biology beyond its canonical role in adaptive immunity [1,7]. Our findings show that MICA and MICB are predominantly found intracellularly in a skin melanoma cell line and in tissue samples from melanoma patients and that they colocalize with major ER chaperones (including CRT, CNX, and ERp57) in a pattern consistent with ER-based folding surveillance [3]. Importantly, we also observe partial CRT-MICA colocalization in normal skin, indicating that this interaction is not exclusive to malignant transformation but likely represents a physiological quality-control checkpoint that becomes quantitatively dysregulated in malignant melanoma.

In skin melanoma biopsies, MICA and MICB displayed marked cytoplasmic accumulation with limited surface staining. This pattern aligns with reports that skin melanoma and other tumors reduce surface NKG2D ligand expression through post-translational mechanisms, including proteasome-dependent degradation of immature MICA, vesicular release, and shedding [9,16,17,18]. Fuertes et al. demonstrated that melanoma cells accumulate EndoH-sensitive, immature MICA in the ER, forming the basis for a potent immune-evasion mechanism driven by aberrant glycosylation [9,10,11]. The cytoplasmic retention observed here is consistent with this model and suggests that defective or incomplete ER maturation contributes directly to reduced immune visibility. Importantly, these alterations do not reflect the emergence of a novel pathway, but rather the quantitative distortion of an existing MHC-related quality-control process.

In BL melanoma cells, confocal microscopy showed perinuclear localization of MICA/MICB with extensive colocalization with CRT, CNX, ERp57, and the ER marker PDI (Supplementary Figure S3) [12,19]. This supports the interpretation that MICA is processed through a canonical chaperone network analogous to, but not identical to, classical MHC-I folding, despite its independence from β_2_-microglobulin and peptide loading [1,6]. These observations reinforce the concept that non-classical MHC molecules share fundamental ER surveillance mechanisms with classical HLA proteins, even though their immunological outputs differ [7,20]. In normal skin, MICA expression was low and primarily observed in basal keratinocytes and adnexal structures, where it partially overlapped with CRT. This partial colocalization in non-transformed tissue strongly suggests that CRT-dependent quality control is a constitutive maturation step, which tumors quantitatively distort to reduce MICA surface availability [9,13].

Biochemical evidence further supports a selective interaction of CRT with immature or underglycosylated MICA. Co-immunoprecipitation experiments revealed two MICA isoforms differing by approximately 7 kDa, consistent with distinct N-glycosylation states [10]. CRT was preferentially associated with the lower-molecular-weight band, consistent with its affinity for monoglucosylated N-glycans, which is typical of incompletely folded glycoproteins. This result mirrors earlier studies showing that altering N-glycosylation via PNGaseF treatment or glycolytic inhibition shifts MICA to faster-migrating forms [10,19]. Together, these data support a model in which CRT transiently binds immature MICA glycoforms, restraining their ER export until proper maturation is achieved [12].

Recombinant protein assays provided additional insight into the molecular basis of this interaction. Using recombinant proteins produced in a prokaryotic system, we observed dose-dependent CRT binding to immobilized MICA by ELISA, indicating that CRT can engage non-glycosylated or structurally immature MICA. In contrast, no detectable interaction was observed by SPR when MICA was expressed in a eukaryotic system, in which recombinant proteins are expected to carry complex N-glycans. These results indicate that the glycosylation state of MICA strongly influences CRT engagement. Rather than implying a stable receptor-ligand interaction, these findings support a checkpoint-like mechanism in which CRT preferentially recognizes immature MICA during early stages of ER maturation [12].

Molecular dynamics simulations support a direct interaction between CRT and MICA. The results reveal a putative binding interface in which both the N- and C-domains of CRT engage the α1/α2 and α3 domains of MICA. These contacts are centered on the lectin region of CRT, although they also involve neighboring non-lectin residues. This binding mode is consistent with prior evidence indicating that the CRT N-domain can associate with non-glycosylated peptides or proteins [21]. At the same time, the C-domain has been reported to participate in diverse protein–protein interactions [22,23]. In this context, the binding pose derived from the modeling aligns well with the literature. While estimates of binding free energies by MM/GBSA should be interpreted with caution, the computational results are generally consistent with experimental data and yield hypotheses that can be tested through structural analysis to guide future experimental validation.

Although our experimental focus was primarily on MICA, we also determined that MICB displayed parallel intracellular localization and ER chaperone colocalization, strongly suggesting that MICB undergoes similar CRT-dependent surveillance. Indeed, MICB shares high structural homology with MICA, including glycosylation sites and intracellular maturation pathways, and displays similar patterns of intracellular retention and post-translational regulation in tumor cells [7], thus supporting the notion that the mechanisms underlying MICA-CRT interaction may extend to MICB. Given the polymorphism of MICA and MICB and the variability of predicted N-glycosylation motifs across alleles, future work should address whether allele-specific glycosylation patterns particular to each ligand modulate the strength or duration of CRT engagement, potentially contributing to inter-individual variability in NKG2D ligand availability on the cell surface.

Besides MICA and MICB, the NKG2D receptor also recognizes MHC-related proteins that belong to the human cytomegalovirus (HCMV) glycoprotein UL-16-binding protein (ULBP) family, which comprises six members (ULBP1-ULBP-6). Expression of these ligands is also induced upon cell stress and tumor transformation, and their surface expression is regulated by post-translational and trafficking mechanisms, leading to NK cell-mediated cytotoxicity on target cells [24,25,26]. ULBPs are widely expressed on cancer cells [27], including melanoma [28,29]. Nevertheless, their subcellular localization differs depending on the ligand. For instance, ULBP2 has been detected on the surface of the fibrosarcoma cell line HT1080, while ULBP5 was mainly found in the cytoplasm of these cells, where it colocalized with CRT [27]. Unlike MICA and MICB, ULBPs lack an α3 domain, suggesting that any interaction with ER chaperones would involve distinct molecular determinants. More studies on the dynamics of ULBPs subcellular location and secretion may shed new light on the immunological surveillance of tumor cells, since ULBPs isoforms and polymorphisms have been shown to determine NKG2D-mediated NK cell function and immune outcomes [30].

Functionally, CRT-dependent retention of MICA provides a unifying framework linking ER stress, aberrant glycosylation, and proteostasis imbalance to impaired NKG2D-mediated immune recognition [13,31]. CRT overexpression in tumors could further enhance this retention, limiting ligand availability at the cell surface. Conversely, acute stress conditions might transiently decrease CRT-mediated retention, increasing MICA surface expression in contexts such as infection or cytokine stimulation. The detection of CRT-MICA colocalization in healthy tissue is particularly informative, as it indicates that this mechanism represents a physiological maturation checkpoint rather than an inherently pathological phenomenon. Melanoma appears to exploit this checkpoint quantitatively, by altering glycosylation, ER stress levels, or chaperone availability, to bias MICA toward intracellular sequestration [13,31].

From a therapeutic point of view, our findings suggest that CRT-dependent retention of MICA represents a potentially druggable immune-evasion checkpoint. On one side, since CRT is a central ER chaperone, its direct targeting would likely compromise global proteostasis; on the other, pharmacological modulation of glycosylation pathways or ER stress responses could reduce CRT-mediated intracellular retention of MICA. In this context, chemical chaperones such as 4-phenylbutyrate or tauroursodeoxycholic acid have been shown to improve protein folding efficiency and ER export under stress conditions, potentially shifting the balance from ER retention toward surface expression of stress-induced ligands [32]. Although these approaches have not been evaluated for MICA, they provide a conceptual framework for how tumor-associated ER stress might be therapeutically manipulated to restore NKG2D ligand expression on the cell surface.

This study has limitations. We did not dissect the contributions of individual MICA N-glycosylation sites (e.g., Asn8, Asn56, Asn102, or the polymorphic Asn211), nor did we examine allele-specific differences such as those observed in MICA*008 [9,11]. In addition, while our data support a mechanistic model of CRT-dependent retention, we did not directly test the functional consequences of CRT modulation on MICA surface expression or NKG2D-mediated effector responses. Future studies combining allele-resolved structural analysis with functional assays, including CRT perturbation and immune activation readouts, will be required to establish causality and physiological impact.

In summary, our results redefine the relationship between MICA and the CRT quality-control cycle. CRT-dependent retention of MICA is best understood as a physiological, glycan-sensitive checkpoint that ensures proper folding before ER export. By revealing how ER proteostasis shapes the availability of a non-classical MHC-I-related immune ligand, this work highlights an additional molecular layer through which MHC-related immune functions are regulated beyond peptide presentation. These findings align with current efforts to understand the molecular physiopathology of MHC/HLA-associated immune regulation and suggest that targeting ER quality-control pathways may represent a strategy to restore immune recognition in melanoma and other MHC-associated diseases.

## 4. Materials and Methods

### 4.1. Patients and Samples

Skin malignant melanoma tissue samples from two patients and skin tissue samples from two healthy individuals were selected from the archives of the Department of Pathology, Hospital de Carabineros (Santiago, Chile). This study was conducted in accordance with the Declaration of Helsinki. All procedures involving human samples were performed in accordance with institutional ethical guidelines.

### 4.2. Cell Lines

The BL melanoma cell line was derived from metastatic lesions of patients treated at Radiumhemmet Hospital, Karolinska [33,34]. BL cells are cataloged in the Cellosaurus database under the accession number CVCL_U802.

The cells were cultured at 37 °C in RPMI 1640 medium supplemented with 5% fetal calf serum (FCS), 1 mM glutamine, 100 IU/mL penicillin, and 100 mg/mL streptomycin (Hyclone, Logan, UT, USA). HEK 293T cells for transfection were maintained in DMEM F12 medium (Hyclone, Logan, UT, USA) supplemented with 5% FCS.

### 4.3. Immunohistochemistry

Paraffin-embedded tissue blocks were cut using the rotational microtome RM2125 (Leica, Wetzlar, Germany) at the Pathology Department of the Clinical Hospital of University of Chile. 4 μm sections were mounted on silanized slides, deparaffinized, and rehydrated through a descending ethanol series to distilled water. Those heavily pigmented melanoma sections underwent melanin bleaching in a 0.05% potassium permanganate solution (Sudelab, Santiago, Chile) for 1 to 2 h, followed by revelation in a 0.1% oxalic acid solution until the color disappeared. The immunohistochemistry procedure was performed as follows: Endogenous peroxidase activity was blocked with 3% hydrogen peroxide for 10 min. After washing with PBS, the sections were incubated with 1% PBS-BSA for 10 min. Without washing, the samples were incubated overnight at 4 °C with the following monoclonal antibodies: anti-MART-1 (Dako-Cytomation, Glostrup, Denmark) as a positive control for melanoma; anti-MICA and anti-MICB (R&D Systems, Minneapolis, MN, USA); and anti-IgG (eBioscience, San Diego, CA, USA) as a control. After three 5-min washes with PBS, the sections were incubated for 15 min with a biotinylated polyclonal rabbit anti-mouse immunoglobulin (KPL, Gaithersburg, MD, USA). The slides were rewashed, and the streptavidin-peroxidase-DAB (Zymed, South San Francisco, CA, USA) revelation system was applied for 15 min. After washing, the slides were incubated with the DAB Plus Substrate Kit (Zymed, South San Francisco, CA, USA) for 5–15 min. The histological preparations were photographed using a Leica microscope with an Olympus C3030 digital imaging system (Olympus, Tokyo, Japan).

### 4.4. Immunofluorescence

Malignant melanoma BL cells (1 × 10^4^) were seeded on round coverslips in 24-well plates and cultured overnight in supplemented RPMI 1640 medium. Cells were fixed with cold methanol (−20 °C, 30 s), washed with PBS, and blocked with 1% PBS-BSA for 15 min at room temperature. Cells were incubated overnight at 4 °C with anti-MICA or anti-MICB antibodies (0.1 ng/mL; R&D Systems, Minneapolis, MN, USA) conjugated to Alexa Fluor 488. Antibodies against calreticulin, calnexin, and ERp57 were conjugated with Alexa Fluor 647 (Molecular Probes, Eugene, OR, USA). Alexa Fluor 488-conjugated IgG2b was used as an isotype control. Nuclei were counterstained with DAPI (300 nM; Thermo Fisher Scientific, Waltham, MA, USA), and coverslips were mounted with Fluoromount-G (Thermo Fisher Scientific, Waltham, MA, USA).

For healthy skin tissue, paraffin-embedded sections were deparaffinized and processed for immunofluorescence following standard procedures. Sections were incubated overnight at 4 °C with a biotinylated goat anti-MICA antibody (BAF1300, R&D Systems, USA) and a rabbit anti-CRT antibody (PA3-900, Thermo Fisher Scientific, Waltham, MA, USA). After washing with PBS and PBS-0.05% Tween 20, sections were incubated for 1 h at room temperature with Alexa Fluor 594-conjugated anti-goat IgG (A-11058) and Alexa Fluor 488-conjugated anti-rabbit IgG (A-11008) secondary antibodies (Thermo Fisher Scientific, Waltham, MA, USA), diluted in PBS containing DAPI, in a humidified chamber protected from light. Following washes, sections were mounted with Fluoromount-G.

Images were acquired using a Zeiss confocal microscope equipped with an LSM5 PASCAL scanning module (FONDAP-CEMC, ICBM, University of Chile) and analyzed using ImageJ software (v1.52v; NIH, Bethesda, MD, USA).

### 4.5. Immunoprecipitation

1 × 10^6^ cells per condition were lysed by adding 200 μL of RIPA buffer pH 7.2 (150 mM NaCl, 10 mM Tris pH 7.4, 5 mM EDTA, 1% Deoxycholic Acid, 0.1% SDS, 1% Triton 100) containing a protease cocktail of 2 ng/mL Pepstatin A, 10 ng/mL Aprotinin, 50 ng/mL Leupeptin, and 1 mM PMSF. The samples were incubated on a rotating platform at 4 °C for 10 min. Cells were then centrifuged at 13,000× *g* at 4 °C for 10 min, and the supernatant was collected and pre-cleared. To the collected supernatant, a tip of Sepharose (Amersham Pharmacia Biotech, Buckinghamshire, UK) along with monoclonal antibody of the same immunoglobulin isotype used for specific immunoprecipitation or rabbit serum without immunization (SNC) when a rabbit polyclonal antibody was used for immunoprecipitation (1 μg/mL), was added. The volume was brought up to 1 mL with PBS. The mixture was rotated at room temperature for 1 h (or overnight at 4 °C). Next, the solution was centrifuged at 2000× *g* for 10 min, and the supernatant was collected. To the supernatant, another tip of Sepharose was added along with the antibody of interest (1 μg/μL). The mixture was rotated at room temperature for 2 h (or overnight at 4 °C). Subsequently, the solution was centrifuged at 1000× *g* for 5 min, the supernatant was discarded, and the precipitate was washed 5 times by adding 1 mL of PBS each time.

### 4.6. Western Blot

The immunoprecipitated fractions were heated at 98 °C for 5 min under reducing conditions. Subsequently, the proteins were separated on a 10% *v*/*v* SDS-PAGE gel using a Mini-PROTEAN III electrophoresis chamber (BioRad, Hercules, CA, USA). A voltage of 50 V was applied to the stacking gel, and 100 V to the running gel. The proteins were then transferred to Hybond-ECL nitrocellulose membranes (Amersham, Buckinghamshire, UK) using a Mini-PROTEAN III horizontal protein transfer system (Bio-Rad, Hercules, CA, USA). A constant current of 100 V was applied for 1 h. The membrane was blocked with 5% *w*/*v* skim milk in PBS overnight at 4 °C. Subsequently, the membrane was washed three times with PBS-Tween for 5 min each, and the primary antibody of interest, diluted in 3% milk-PBS (1:500), was added and incubated at room temperature for 2 h. The membrane was washed seven times with PBS-Tween. Next, the secondary antibody conjugated with HRP, diluted in milk-PBS (1:500), was added. The membrane was washed five times with PBS-Tween. Finally, the membrane was incubated with ECL Western Blotting Substrate (Pierce, Rockford, IL, UK), then placed on a photographic developing tray with D-76 developer and E-6 fixer (Kodak, Rochester, NY, USA), and exposed to X-ray film (Pierce, Rockford, IL, USA) for subsequent chemiluminescence visualization.

### 4.7. Recombinant Prokaryotic MICA and CRT

The ectodomains of human MICA (α1, α2, α3 domains) were cloned into the pETb15 vector (Novagen, Madison, WI, USA). Human CRT, coding for residues 18–417, was cloned into the pET-15b vector (Novagen, Madison, WI, USA). Both vectors were transformed in *E. coli* BL21 DE3 and purified in agarose Ni-NTA columns (Invitrogen, Carlsbad, CA, USA) and via anion exchange chromatography (UNO Q-6 Biochromatography column, Bio-Rad, Hercules, CA, USA), as previously described [35,36].

### 4.8. ELISA

A 96-well flat-bottom Maxisorp plate (Nunc, Roskilde, Denmark) was coated overnight at 4 °C with 100 μL per well of 0, 3, 6, and 12 μg/mL of recombinant human MICA protein. Subsequently, the plate was washed five times with 1% BSA-PBS-0.1% Tween 20 and further blocked with 200 μL of 1% BSA-PBS-Tween per well for 1 h at 37 °C. After stopping, 100 μL of recombinant human CRT at concentrations of 0, 3, 6, and 12 μg/mL was added in triplicate wells. The plate was incubated for 2 h at 37 °C. Next, a solution of polyclonal rabbit anti-human CRT antibody at a dilution of 1:2500 (0.025 ng/mL) was added to each well at 100 μL and incubated for 1 h at 37 °C. After five washes with 1% BSA-PBS-Tween, 100 μL per well of HRP-conjugated anti-rabbit antibody at a 1:500 dilution (0.5 μg/mL) was added and incubated for 1 h at 37 °C. The HRP activity was evaluated by adding the TMB Substrate Solution (Thermo Fisher Scientific, Waltham, MA, USA). The optical density was read at 450 nm using an automated ELISA reader (Bio-Rad, Hercules, CA, USA).

### 4.9. Recombinant Eukaryotic MICA

The pCMV-SPORT6-MICA vector (Clone ID 4446575) and the pHLSec-Avitag destination vector [37] were employed in this study. The vectors were digested using KpnI and AgeI restriction enzymes (New England Biolabs, Ipswich, MA, USA). The digestion reactions were carried out at 37 °C for 4 h, followed by incubation at 65 °C for 20 min and cooling to 4 °C overnight. Subsequently, ligation reactions were performed at a 3:1 molar ratio of insert to vector. A final volume of 10 μL containing 3 units of Quick Ligase and 2X buffer (New England Biolabs, Ipswich, MA, USA) was used for the ligation reaction, which was conducted at room temperature for 5 min. For transfection, HEK293T cells at 90% confluence were used. A total of 5 µg of plasmid DNA dissolved in 2 mL of DMEM F-12 medium (Invitrogen, Carlsbad, CA, USA) was required for transfection. The transfection was performed on one million HEK-293 cells using Lipofectamine (Invitrogen, Carlsbad, CA, USA), and the cells were cultured for 4 days under standard conditions in DMEM medium. At the end of the incubation, a Western blot analysis with anti-His-Tag antibodies was performed to assess transfection efficiency in both total cell lysates and culture supernatants. The positive transfection supernatants were quantified using a NanoDrop spectrophotometer (NanoDrop, Wilmington, DE, USA) for subsequent BIAcore studies.

### 4.10. Surface Plasmon Resonance (SPR)

The ligand preparation was carried out as follows: 2 mL of supernatant from the transfected HEK293T cell culture was diluted with 4 mL of PBS. 400 μL of Talon resin was added, and the mixture was incubated for 24 h with agitation at 4 °C. The following day, the resin was washed with 10 mM Tris (pH 8.0) and resuspended in 500 μL of 10 mM Tris pH 8.0. The mixture was transferred to a 1.5 mL tube, and 100 μL of the red component from the Biotin Protein Ligase Kit (Avidity, Aurora, CO, USA) and 100 μL of the blue component were added, followed by incubation for 24 h on an orbital shaker at 4 °C. The resin was washed in a disposable plastic column (Millipore, Burlington, MA, USA) with PBS containing 10 mM Tris (pH 8.0). Elution was performed with 300 mM imidazole in 10 mM Tris (pH 8.0) PBS diluted in HEPES pH 7.4, 150 mM NaCl. The eluate was collected in fractions of up to 1 mL. After measuring the concentration using a nano-spectrophotometer (Nanodrop, USA), the eluate was diluted to a final concentration of 1 μg/μL in Tris buffer pH 8.0. The BIAcore Sensor Chip CM5 (BIAcore, Uppsala, Sweden) was conjugated with a concentration of 1 μg/μL of recombinant MICA protein obtained in 10 mM HEPES pH 7.5, 150 mM NaCl, 0.05% Tween buffer, with a maximum sample volume of 200 μL. The analyte (recombinant CRT) was applied at concentrations of 1 nM, 5 nM, and 20 nM using the BIAcore 2000 instrument (BIAcore, Uppsala, Sweden). The data were obtained using BIAcore T100 version 2.0 software (BIAcore, Uppsala, Sweden).

### 4.11. In Silico Binding Prediction

The structural models of CRT and MICA were generated using the SWISS-MODEL server [38] with their available crystal structures as templates (PDB IDs 7QPD and 1HYR, respectively) [39,40]. ClusPro2.0 [41] was employed to perform global protein-protein docking. The 3 top complexes with the biggest clusters resulting from the worldwide docking were selected for molecular dynamics. The 3 chosen complexes were solvated with a TIP3P water box and NaCl at 0.15 M using the CHARMM-GUI server [42]. The FF19SB force field [43] was employed for all molecular dynamics simulations.

The following molecular dynamics simulation protocol was employed, utilizing the AMBER20 [44] suite of programs. This entailed an initial minimization step using up to 5000 conjugate gradient steps, followed by 2500 steepest descent steps. Subsequently, a heating step was conducted from 0 to 310 K in an NPT assembly for 100 ps. This was achieved using a harmonic restraint of 10.0 kcal mol^−1^ Å^−2^ in all atoms of the proteins. Subsequently, the system was subjected to a 1 ns equilibration step without constraints, allowing it to reach equilibrium. Additionally, 500 ns production runs were conducted to calculate binding energy using MMPBSA.py [45] to select a stable CRT-MICA complex conformation for subsequent analysis.

## 5. Conclusions

Calreticulin emerges as a key ER quality-control component regulating the maturation of the non-classical MHC-I molecule MICA. Across skin malignant melanoma tissues and the BL melanoma cell line, MICA/MICB shows predominant intracellular retention and colocalization with ER chaperones, supporting ER-based surveillance of NKG2D ligands. Biochemical assays indicate that CRT preferentially associates with a lower-molecular-weight variant, likely an underglycosylated form of MICA, while recombinant protein experiments and modeling support a direct interaction that is favored when MICA lacks complex N-glycans. Collectively, our findings support a checkpoint-like model in which CRT engages immature MICA during early ER maturation, potentially limiting surface availability and contributing to reduced NKG2D-mediated immune recognition in melanoma. Future work dissecting allele- and site-specific glycosylation requirements and testing CRT perturbation on MICA surface expression and effector activation will be essential to establish causality and therapeutic relevance.

## Data Availability

The data supporting the findings of this study are included within the article and its Supplementary Materials. Additional data are available from the corresponding author upon reasonable request.

## Supplementary Materials

The following supporting information can be downloaded at: https://www.mdpi.com/article/10.3390/ijms27031310/s1.

## Abbreviations

The following abbreviations are used in this manuscript:

MICA	Major histocompatibility complex class I chain-related gene A
MICB	Major histocompatibility complex class I chain-related gene B
CRT	Calreticulin
CNX	Calnexin
ER	Endoplasmic reticulum
NKG2D	Natural Killer Group 2D
MHC-I	Major Histocompatibility Complex class I
ERp57	Endoplasmic Reticulum Protein 57
HLA	Human Leukocyte Antigen
HE	Hematoxylin and eosin
SPR	Surface Plasmon Resonance
MD	Molecular Dynamics
MART-1	Melanoma Antigen Recognized by T-cell 1
NK cell	Natural Killer cell
HEK	Human Embryonic Kidney cell
BL	Melanoma cell line
ELISA	Enzyme—Linked Immunosorbent Assay
Endo-H	Endoglycosidase H
Rg	Radius of Gyration
RMSD	Root Mean Square Deviation
RMSF	Root Mean Square Fluctuation
DCCM	Dynamic Cross-Correlation Matrix
MM/GBSA	Molecular Mechanics/Generalized Born Surface Area
HRP	Horseradish Peroxidase
PDI	Protein Disulfide Isomerase
DAB	3,3′-Diaminobenzidine
DAPI	4′,6-Diamidino-2-Phenylindole
FCS	Fetal Bovine Serum
BSA	Bovine Serum Albumin
PBS	Phosphate-Buffered Saline
SDS-PAGE	Sodium Dodecyl Sulfate-Polyacrylamide Gel Electrophoresis
TMB	3,3′,5,5′-Tetramethylbenzidine
Ni-NTA	Nickel-Nitrilotriacetic Acid
IgG	Immunoglobulin G
TIP3P	Transferable Intermolecular Potential 3-point water model
NPT	Constant Number of particles, Pressure and Temperature ensemble
PNGaseF	Peptide-N-Glycosidase F
PMSF	Phenylmethylsulphonyl fluoride
SNC	Normal Rabbit Serum
RS	Preimmune Rabbit serum
ECL	Enhanced Chemiluminescence
