# Supplementary material for: Calreticulin-Mediated Quality Control of the Non-Classical MHC-I Molecule MICA: Implications for Immune Surveillance
Source: Int J Mol Sci. 2026 Jan 28;27(3):1310. doi: 10.3390/ijms27031310 (PMC12897870; doi:10.3390/ijms27031310)
Supplement: Supplementary file 1 [file ijms-27-01310-s001.zip › ijms-4101681-supplementary.pdf]

Supplementary Figures

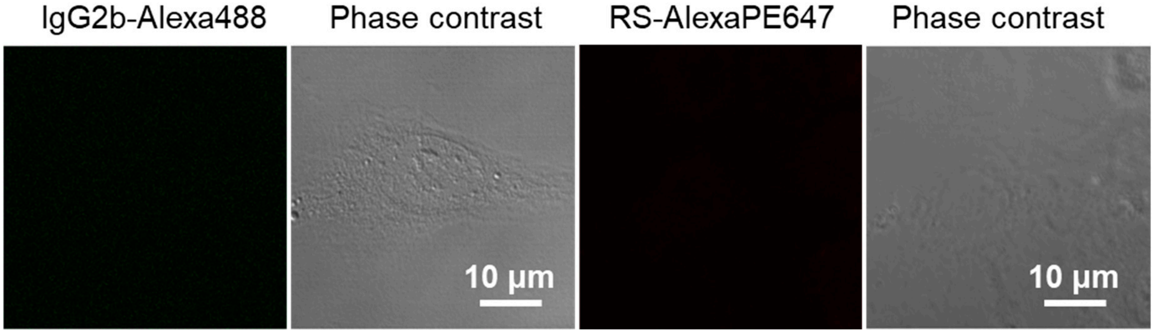

Supplementary Figure S1. Immunofluorescence isotype controls in BL melanoma cells. BL melanoma cells were incubated with an Alexa Fluor 488-conjugated IgG2b antibody and rabbit preimmune serum (RS) conjugated to Alexa Fluor 647, used as isotype and negative controls for immunofluorescence staining. No specific fluorescence signal was detected in either channel. Corresponding phase-contrast images are shown to indicate cell morphology. Scale bars: 10 μm.

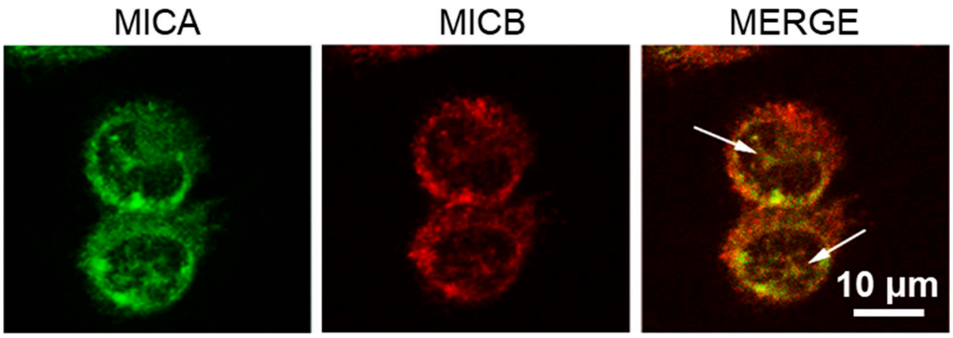

Supplementary Figure S2. Localization patterns of MICA and MICB in BL melanoma cells. Representative confocal immunofluorescence images of BL melanoma cells showing intracellular staining of MICA (green) and MICB (red). The merged image indicates regions of signal overlap (yellow; white arrows), consistent with a shared intracellular/perinuclear distribution. Scale bar: 10 μm.

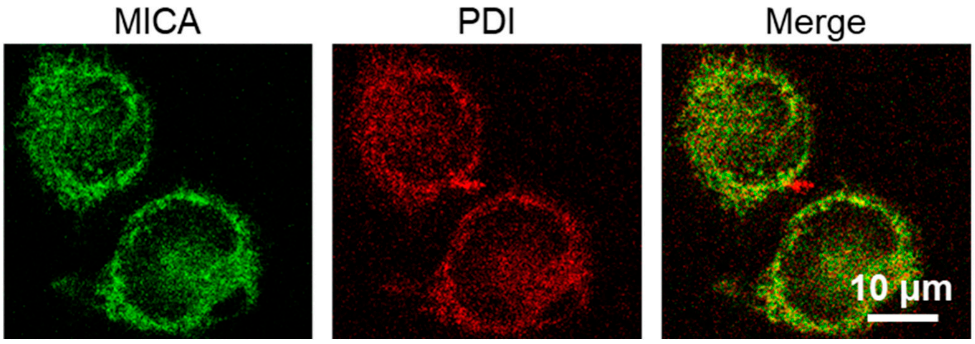

Supplementary Figure S3. Colocalization of MICA with the ER marker protein disulfide isomerase (PDI) in melanoma cells. Confocal immunofluorescence images of BL melanoma cells showing intracellular localization of MICA (green) and protein disulfide isomerase (PDI; red). The merged image (yellow) indicates partial colocalization, consistent with endoplasmic reticulum localization of MICA. Scale bar: 10 µm.

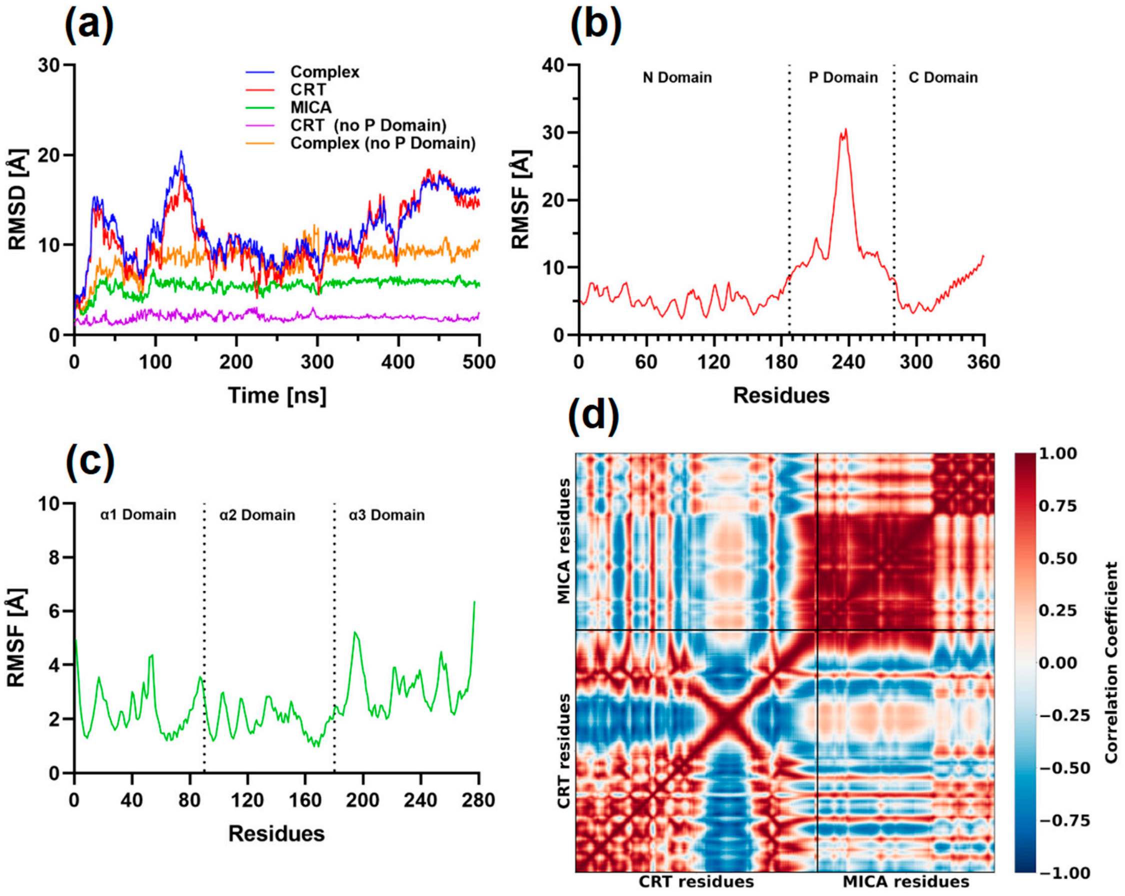

Supplementary Figure S4. Structural stability of the MICA-CRT complex throughout the molecular dynamics. a) RMSD as a function of time for the global complex (blue), CRT (red), MICA (green), and their structured cores excluding the CRT P-domain (purple and orange). (b) RMSF of CRT, highlighting the compartmentalized dynamics of the N, P, and C domains. The P-domain (residues 190-280) displays significant conformational plasticity, peaking at ~30 Å. (c) RMSF of MICA, showing the local fluctuations of the $\alpha1$, $\alpha2$, and $\alpha3$ domains. (d) DCCM illustrating the normalized covariance of atomic displacements. Red regions indicate correlated motions, while blue regions indicate anti-correlated motions, highlighting the mechanical coupling between the CRT core and the MICA interface residues.
